# Neurotoxicity and Cytokine Release Syndrome After Chimeric Antigen Receptor T Cell Therapy: Insights Into Mechanisms and Novel Therapies

**DOI:** 10.3389/fimmu.2020.01973

**Published:** 2020-08-28

**Authors:** Elizabeth L. Siegler, Saad S. Kenderian

**Affiliations:** ^1^T Cell Engineering, Mayo Clinic, Rochester, MN, United States; ^2^Division of Hematology, Mayo Clinic, Rochester, MN, United States; ^3^Department of Immunology, Mayo Clinic, Rochester, MN, United States; ^4^Department of Molecular Medicine, Mayo Clinic, Rochester, MN, United States

**Keywords:** CART, chimeric antigen receptor, immunotherapy, CRS, neurotoxicity

## Abstract

Chimeric antigen receptor T (CART) cell immunotherapy has been remarkably successful in treating certain relapsed/refractory hematological cancers. However, CART cell therapy is also associated with toxicities which present an obstacle to its wider adoption as a mainstay for cancer treatment. The primary toxicities following CART cell administration are cytokine release syndrome (CRS) and immune effector cell-associated neurotoxicity syndrome (ICANS). New insights into the mechanisms of these toxicities have spurred novel treatment options. In this review, we summarize the available literature on the clinical manifestations, mechanisms, and treatments of CART-associated CRS and ICANS.

## Introduction

Three mainstays of cancer therapy – surgery, chemotherapy, and radiation – are often insufficient to induce long-term remissions. Within the last few decades, cancer immunotherapy has made great strides in harnessing and enhancing the patient’s own immune system to fight cancer. One such type of cancer immunotherapy is chimeric antigen receptor T (CART) cell therapy, in which T cells are engineered to express a synthetic receptor which confers specificity against a particular antigen overexpressed on the cancer cell surface. Since the late 1980s, CART cells have gone from a little-known academic project (1) to an FDA approved therapy which has treated over 1000 patients with cancer in the United States alone.

Initially, CARs consisted of an antibody-derived extracellular antigen recognition domain linked to an intracellular CD3ζ signaling domain. These first-generation CART cells showed little therapeutic effects in early clinical trials (2–5). A major breakthrough in the field occurred when researchers developed second-generation CART cells, which included an additional costimulatory domain such as 4-1BB or CD28 to improve persistence and potency.

In the last decade, second-generation CART cells have led to great clinical success in treating B-cell malignancies. The first promising CART clinical trial showed durable remission in a patient with chronic lymphocytic leukemia treated with CD19-targeted CART (CART19) cells (6). Other early clinical trials showed similar potential in treating B-cell cancers (7, 8). These early successes paved the way for pivotal trials, such as the ELIANA trial for pediatric patients with acute lymphoblastic leukemia (ALL) (9) and the ZUMA-1, JULIET, and TRANSCEND trials for patients with B-cell lymphomas (10–12). CART pivotal trials demonstrated astounding overall response rates (ORR) and complete responses (CR) and led to the FDA approval of two CART products, tisagenlecleucel and axicabtagene ciloleucel, in 2017.

Despite the astonishing initial success in treating many B-cell malignancies, CART cell therapy often fails to yield durable responses. Although ORR and CR are generally high in CART cell clinical trials against blood cancers, 30–60% of patients will relapse (13) due to limited CART persistence (14) or antigen escape (15, 16). These relapse mechanisms have been observed in both blood cancers and solid tumors following CART cell treatment. Additionally, solid tumors are surrounded by an immunosuppressive tumor microenvironment composed of immune cells, vasculature, and stromal cells, which create physical and immunological barriers to CART cells. As such, clinical trials of CART cell therapy in solid tumors have been largely disappointing.

In addition to a lack of antitumor efficacy or durable remission, severe toxicities following CART cell administration pose significant threats to patients. Despite remarkable clinical success, incidences of CART-associated toxicities remain high. These toxicities are often severe and occasionally fatal. One analysis of over 1000 patients treated with tisagenlecleucel or axicabtagene ciloleucel reported the sobering statistic that 7% of patients died due to non-relapse mortality within 30 days of initial CART cell administration (17). Life-threatening adverse events present a challenge to more widespread adoption of CART cell therapy in the clinic. In this review, we will discuss types and mechanisms of the two most common CART-associated toxicities, cytokine release syndrome (CRS) and neurotoxicity, as well as existing and emerging management strategies.

## Cytokine Release Syndrome

### Clinical Manifestations

The most common toxicity after CART cell infusion is CRS, which has been reported to be as high as 100% in some CART19 clinical trials (9, 10, 18–20). Various grading scales have been used in clinical trials by different groups to determine the severity of CRS; consequently, the reported rates of CRS vary among the different scales. The American Society for Transplantation and Cellular Therapy (ASTCT) created a grading scale in order to standardize the assessment and treatment of CART-associated toxicities, including CRS. CRS of any severity can include constitutional symptoms such as headache, nausea, dyspnea, myalgias, and malaise. According to ASTCT guidelines, Grade 1 CRS includes fever (≥38.0°C) without hypotension or hypoxia. Grade 2 CRS is described as fever plus hypotension without requiring vasopressors and/or hypoxia requiring oxygen via low-flow nasal cannula. Grade 3 CRS presents with fever, hypotension requiring one vasopressor, and hypoxia requiring high-flow nasal cannula. Finally, Grade 4 CRS escalates to fever plus hypotension requiring multiple vasopressors and/or hypoxia requiring positive pressure (21).

Acquired hemophagocytic lymphohistiocytosis/macrophage activation syndrome (HLH/MAS) also stems from immune hyperactivation. Patients who develop HLH/MAS present with fever as well as elevated serum levels of ferritin, triglycerides, and cytokines including interferon (IFN)γ, interleukin (IL)-6, IL-10, macrophage inflammatory protein (MIP)1β, and monocyte chemoattractant protein (MCP)-1 (22, 23). Most patients with severe CRS display laboratory markers that meet the criteria for HLH/MAS, and in most cases, HLH/MAS symptoms fade with CRS resolution (24), indicating that these two hyperimmune disorders share common pathways and features and are not easily untangled from each other. As such, HLH/MAS is not treated as a separate CART-associated adverse event according to ASTCT guidelines.

Cytokine release syndrome onset typically occurs during the first week after CART cell treatment. However, patients treated with 4-1BB-costimulated CART cells often experience later onset of CRS than patients treated with CD28-costimulated CART cells (25). CRS onset usually coincides near the peak of CART cell expansion and cytokine production. In general, CD28-costimulated CART cells expand more rapidly than slower growing but more persistent 4-1BB-costimulated CART cells (26–28), contributing to the difference in CRS onset between these two constructs.

C-reactive protein (CRP) and ferritin are diagnostic markers of CRS and are monitored daily after CART cell infusion (29). Elevated D-dimer and low fibrinogen are indicative of coagulopathy following CART cell therapy and are also checked daily (30). Elevated triglycerides are a symptom of HLH/MAS and are monitored post-CART cell infusion as well (29). In addition to these clinical markers, several predictive biomarkers of CRS have been proposed. These predictive markers may lead to preemptive treatment in high-risk patients; preliminary data of early intervention in pediatric patients with mild CRS demonstrated trends toward reducing the incidence of subsequent severe (Grade 3+) CRS (31). Scientists reported a predictive algorithm of CRS in pediatric patients based on serum levels of IFNγ, IL-13, and MIP1α within 72 h after CART cell infusion (24). Researchers found that cytokines including IFNγ, IL-6, IL-8, IL-10, and MCP-1 were significantly higher within 36 h of CART cell administration in patients who went on to experience severe (Grade 4+) CRS compared to patients who developed mild or moderate CRS. They found that, in particular, elevated MCP-1 levels above a certain threshold were the most accurate in predicting severe CRS development. These researchers also identified several independent predictors of CRS: high tumor burden, prior lymphodepletion with fludarabine and cyclophosphamide, high CART dose, and high peak CART blood counts all correlated with CRS development (32). Some of these risks can be mitigated, for example, by debulking the tumor prior to CART cell infusion (22, 33, 34). However, many of the factors that contribute to a higher risk of CRS also help optimize CART cell efficacy. Lymphodepleting regimens such as fludarabine/cyclophosphamide are associated with greater CRS occurrence, yet are important in creating a favorable immune environment for CART cell activity. Fludarabine/cyclophosphamide conditioning has been shown to influence the cytokine milieu, possibly by eliminating cells that serve as cytokine sinks for IL-7 and IL-15 and subsequently increasing the availability of these pro-survival cytokines to therapeutic lymphocytes, in addition to eliminating immunosuppressive cells such as regulatory T cells (29, 35–37). Combination of CART cell therapy with checkpoint blockade has displayed evidence of boosting CART cell efficacy but also has the potential to increase the risk of CRS (38, 39), although some clinical trials have not reported excess toxicity (40, 41). Furthermore, although it is an undesired and occasionally severe side effect, the occurrence of CRS is also linked to improved clinical response to CART cell therapy.

It is important to note that while most clinical experience involves CART19 cell therapy, CART cell therapies targeting other hematological antigens such as BCMA have resulted in CRS incidence at comparable levels to CART19 cells (42). CRS-like symptoms, including fever, lymphopenia, myalgia, and headache, were detailed in a case study of a patient who received intracranial IL-13Rα2-targeted CART cell infusions for glioblastoma (43); however, these symptoms were mild, and the localized routes of administration did not appear to increase unwanted toxicity. In a phase I trial of EGFRvIII-targeted CART cells given intravenously to patients with glioblastoma, symptoms including fever, hypotension, and elevated CRP and IL-6 were observed but attributed to localized intracranial CRS rather than systemic CRS (44). Other than these isolated observations during the treatment of glioblastoma, CRS has not been reported in CART cell therapy for solid tumors to date. CRS results from massive CART cell activation and ensuing systemic inflammation; CART cells are more localized and less stimulated in solid tumors than in hematological cancers and as such, do not trigger CRS. Rather, the main CART-associated toxicity in solid tumors is on-target off-tumor effects, in which CART cells attack not only tumor cells but also healthy cells expressing the target antigen. On-target off-tumor toxicity has been noted in clinical trials of CART cells targeting CAIX (45), Her2 (46, 47), CEA (48), IL13Rα2 (49), EGFR (50), EGFRvIII (51), CD171 (52), and TAG-72 (53).

### Mechanisms

**FIGURE 1:**
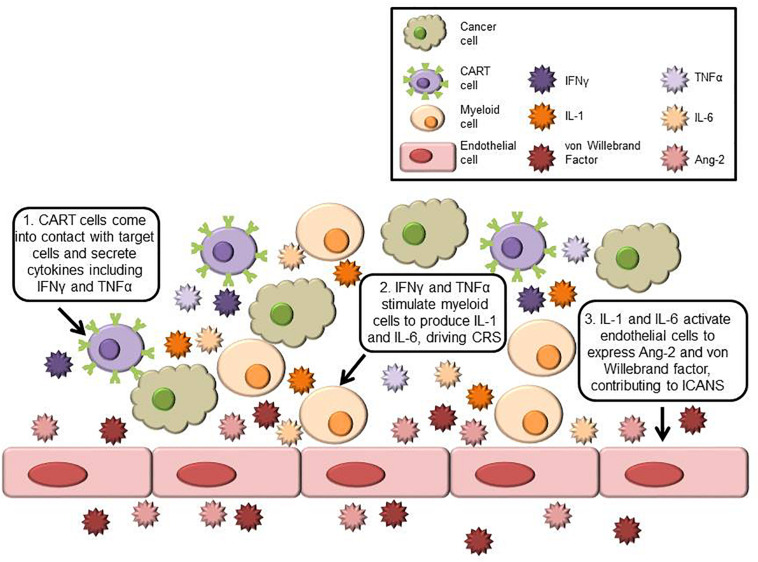
Schematic of key cells and cytokines involved in CRS and ICANS. Cancer cells expressing the target antigen stimulate CART cells to secrete inflammatory cytokines including IFNγ and TNFα. These cytokines activate myeloid cells, which produce CRS-linked cytokines such as IL-1 and IL-6. In ICANS, activated endothelial cells produce von Willebrand factor and Ang-2 and contribute to blood-brain barrier dysfunction.

Greater insight into the mechanisms behind CART-associated toxicities has been made through correlative science and retrospective analysis of clinical trials and through improvements in preclinical models. Key cells and cytokines involved in CART-related toxicities are shown in Figure 1, and symptoms, markers, and mechanisms are summarized in Table 1. Mechanisms behind CRS are complicated and poorly understood. After the patient is infused, CART cells come into contact with target cells and release inflammatory cytokines such as tumor necrosis factor (TNF)α and IFNγ, which in turn activate monocytes and macrophages to secrete cytokines including IL-1, IL-6, and inducible nitric oxide synthase (iNOS).

**TABLE 1:** Summary of CART cell toxicities.

CART-associated toxicity	Symptoms	Diagnostic criteria	Laboratory markers	Main Source of laboratory markers	Mechanism	References
CRS	Fever, headache, nausea, dyspnea, myalgias, malaise, capillary leak, multiorgan dysfunction	Fever; hypotension; hypoxia; elevated CRP, ferritin, D dimer, fibrinogen, triglycerides	IFNγ TNFα GM-CSF IL-10 IL-1 IL-6 iNOS	T cells T cells T cells Myeloid cells Myeloid cells Myeloid cells	Activated CART cells provoke inflammatory response from myeloid cells	(54) (10) (20)
ICANS	Aphasia, tremor, dysgraphia, lethargy, obtundation, stupor, seizures, coma	ICE score, depressed level of consciousness, seizure, motor findings, cerebral edema	IL-1 IL-6 IL-8 IP-10 MCP-1 Quinolinic acid VEGF Von Willebrand factor Ang-2 CD14 + cells in CSF	Myeloid cells/microglia Myeloid cells/microglia, pericytes Myeloid cells/microglia Myeloid cells/microglia Myeloid cells/microglia Myeloid cells/microglia Pericytes Endothelial cells Endothelial cells Myeloid cells	Activated CART cells provoke inflammatory response from myeloid cells, systemic inflammation activates endothelial cells and drives blood-brain barrier dysfunction	(82) (81) (22, 89)

Recent studies are shedding more light on the cytokines secreted by myeloid cells in relation to CRS development. The inflammatory cytokine IL-6 is highly elevated in patients experiencing CRS (54), indicating an important role in mediating CRS onset. Preclinical models of CRS have shown that engrafted human CART cells produce IFNγ and granulocyte-macrophage colony-stimulating factor (GM-CSF), which in turn activate myeloid cells. Host myeloid cells release large amounts of IL-6 and iNOS when colocalized with tumor and CART cells. Furthermore, engrafting mice with CART cells expressing murine CD40L, which interacts with host myeloid cells, exacerbates CRS symptoms (55). These data suggest that direct interactions between CART and host myeloid cells – primarily macrophages – lead to increased IL-6 and iNOS production and subsequent CRS; however, earlier *in vitro* studies have concluded that direct contact between CART and myeloid cells is not required to produce IL-6 (56). Animal models have also shown a correlation between monocyte count and CRS mortality after CART cell administration. Monocytes were found to be the primary source of IL-1, which preceded monocyte production of IL-6. Depleting monocytes prior to CART cell treatment decreased CRS incidence but also dampened CART cell therapeutic effects (57). Many HLH/MAS symptoms and markers overlap with those of severe CRS; the association of HLH/MAS with CRS also points to the importance of macrophage hyperactivation as a trigger of CRS.

### Treatments

Elucidating the mechanisms of CART-related toxicities has facilitated the development of more effective treatment protocols and of novel treatment approaches. Table 2 summarizes the current and investigational approaches for the treatment of CART-associated toxicities. Treatment plans can be guided by the ASTCT grading system for CART toxicities (21). Current guidelines for CRS management after CART cell therapy vary between clinics but typically involve supportive care and treatment with the anti-IL-6R antibody, tocilizumab. Originally used to treat rheumatoid and juvenile arthritis, tocilizumab was FDA approved alongside tisagenlecleucel in 2017 to treat CRS after CART cell therapy (9, 10). Tocilizumab does not appear to affect CART cell efficacy in mice (57) or therapeutic outcomes in patients (10, 19, 58, 59). Tocilizumab often resolves symptoms of CRS within hours and has become the standard of care. Siltuxumab is a clinically available antibody against IL-6 and has also been used to treat CRS, although less frequently than tocilizumab (60). Corticosteroids have been used to treat severe CRS if unresponsive to tocilizumab (7, 10, 33, 61).

**TABLE 2:** Summary of current and investigational approaches to CART-associated toxicities.

Treatment strategy	Therapeutic agent	Rationale	Stage	Clinical trial identifier	References
IL-6 or IL-6R inhibition	Siltuxumab, tocilizumab	IL-6 highly elevated during CRS, produced by activated myeloid cells, key cytokine in CRS development	Clinical, standard of care (tocilizumab)	–	(9) (10) (58) (19)
Corticosteroids	Dexamethasone, methylprednisolone	Immunosuppression to quiet overactive immune cells	Clinical, standard of care	–	(10) (18, 61) (33) (7)
GM-CSF depletion	Lenzilumab, GM-CSF gene knockout	GM-CSF involved in stimulation of myeloid cells, which are implicated in CRS and ICANS	Preclinical, clinical trial initiated	NCT04314843	(62) (63)
IL-1 inhibition	Anakinra	IL-1 elevated during ICANS, produced by activated myeloid cells, precedes IL-6 secretion	Preclinical, clinical trial initiated	NCT04148430, NCT04150913	(57) (55)
TNFα inhibition	Etanercept	TNFα elevated during CRS, produced by activated CART cells, key cytokine in CRS development	Clinical trials ongoing	NCT03050190	(67) (68) (69) (70)
JAK/STAT inhibition	Ruxolitinib, itacitinib	JAK/STAT pathway utilized by IL-6 and GM-CSF	Preclinical, clinical trial ongiong	NCT04071366	(72) (73) (74)
ITK inhibition	Ibrutinib	Retrospective analysis showed patients previously treated with ibrutinib had improved CART cell therapeutic outcomes, ITK inhibition dampens inflammatory cytokines but enhances Th1 functions	Clinical trials ongoing	NCT02640209, NCT03960840, NCT01865617, NCT04234061, NCT03331198, NCT03310619	(75) (77) (78) (79) (80)
Pharmacological T cell activation switch	Dasatinib	T-cell receptor kinases utilized in CART cell signaling, reversibly inhibited to dampen immune overactivation	Preclinical	–	(98) (99)
Endothelial cell protection	Defibrotide	Endothelial cell activation from systemic inflammation a key driver of ICANS	Clinical trial initiated	NCT03954106	(92)
Suicide genes and selection markers	Inducible caspase 9, truncated EGFR, CD20	CART cells selectively ablated if dangerously overactivated	Clinical trials ongoing	NCT02107963, NCT01822652, NCT03373071, NCT03618381, NCT03084380, NCT02937844, NCT03710421, NCT02159495, NCT02844062	(93, 94, 95, 96, 97)

Patients refractory to tocilizumab and corticosteroids remain difficult to treat, and new strategies for the management or prevention of CRS are ongoing. Given the success of tocilizumab in treating CRS, there is a strong rationale for the selective inhibition of additional key cytokines involved in CART-related toxicities. GM-CSF has been implicated in the stimulation of myeloid cells, which greatly contribute to CRS development. Preclinical studies have shown that treatment with the anti-GM-CSF antibody, lenzilumab, had no negative effects on CART cell function *in vitro* or *in vivo* and even improved leukemic disease control in mice. Furthermore, GM-CSF neutralization reduced CRS symptoms in a patient-derived xenograft model. GM-CSF was also genetically nullified by using a CRISPR-Cas9 platform; GM-CSF knockout CART cells led to improved overall survival in mice, indicating additional potential for next-generation gene-edited CART cells (62). Another study demonstrated that GM-CSF neutralized by antibodies or knocked out with TALEN technology ablated macrophage-associated cytokines linked to CRS development, including MCP-1, IL-6, and IL-8 (63). A clinical trial has been designed using lenzilumab to prevent toxicities in patients receiving axicabtagene ciloleucel.

IL-1 is an inflammatory cytokine produced by myeloid cells and has been linked to CRS. Anakinra, another drug used to treat rheumatoid arthritis, is an IL-1R antagonist and has been explored to treat CART-associated toxicities. Researchers found that monocytes produced IL-1 earlier than IL-6 when cocultured with CART cells. When mice were treated with anakinra, CRS was eliminated while CART cell anticancer efficacy was preserved (57). In another preclinical study, anakinra downregulated iNOS expression by macrophages and reduced mortality due to CRS in CART-treated mice (55). Anakinra has been shown to be effective in treating patients with HLH (64–66), and clinical trials have been initiated to investigate this promising strategy for CART-related CRS.

Treatment with the soluble TNFα receptor, etanercept, helped rapidly resolve CRS symptoms in one pediatric patient (67) but had no clear clinical benefit in an adult patient (68), both of whom experienced severe CRS after CART19 cell infusion. However, etanercept is more widely used to treat CART-associated CRS in clinical trials in China: several patients were treated with etanercept alone or in combination with tocilizumab during phase I/II trials (69, 70).

Another approach to managing CRS is to modulate the T-cells with small molecule inhibitors. IL-6 and GM-CSF utilize the JAK/STAT signaling pathway, and inhibiting this pathway has shown to be effective at dampening CRS after CART cell treatment. Ruxolitinib is an FDA approved JAK/STAT pathway inhibitor which has been shown to reduce inflammatory cytokines in preclinical studies and clinical trials for myeloproliferative neoplasms (71). Ruxolitinib diminished inflammatory cytokines such as IFNγ and TNFα, alleviated symptoms of CRS, and prolonged overall survival in a mouse model of CART-induced CRS (72). However, non-specific targeting of the JAK/STAT pathway might be detrimental to T-cell functions. JAK-1 inhibitors have been investigated as well: itacitinib exerted greater control over inflammatory cytokines than tocilizumab *in vitro* and reduced serum levels of CRS-linked cytokines without impacting CART cell function *in vivo* (73). An ongoing phase II clinical trial is investigating itacitinib for the prevention of CRS in patients treated with CART cells (74).

Bruton’s tyrosine kinase (BTK) is a critical component in B-cell receptor signaling, and the BTK inhibitor, ibrutinib, is FDA approved to treat B-cell chronic lymphocytic leukemia. Researchers found that patients treated with ibrutinib for at least 1 year prior to CART cell infusion had decreased T-cell exhaustion markers, better CART cell expansion, and improved clinical outcomes (75). These observations spurred investigation into the effects of ibrutinib on CART cell therapy. Ibrutinib also inhibits IL-2 inducible T-cell kinase (ITK), a tyrosine kinase in the same family as BTK, and appears to enhance Th1 functions by preferentially inhibiting Th2 CD4 T-cells (76). Animal models also demonstrated that combination therapy of ibrutinib with CART cells was more effective than either therapy alone, and CART cells administered with ibrutinib expressed lower levels of exhaustion marker PD1. Peripheral T-cell and CART cell counts were also increased in ibrutinib-treated mice, possibly due to inhibition of the CXCR4 pathway and subsequent peripheral blood lymphocytosis (77). Ibrutinib was further found to drastically reduce IL-6, IFNγ, TNFα, and GM-CSF, preventing CRS and prolonging survival in CART-treated mice. ITK inhibition by ibrutinib is likely the mechanism behind the observed decrease in T-cell inflammatory cytokines, and ibrutinib has the potential to both enhance efficacy and improve safety of CART cell therapy (78). Clinical trials of combination ibrutinib and CART cell therapy are ongoing, and initial results are promising (79, 80).

## Neurotoxicity

### Clinical Manifestations

Neurotoxicity, also referred to as immune effector cell-associated neurotoxicity syndrome (ICANS), is another common and unique toxicity following CART cell therapy, occurring in up to 67% of patients with leukemia and 62% of patients with lymphoma (81). ICANS usually appears within one to 3 weeks after CART cell infusion, although there have been reports of delayed ICANS development. ICANS often accompanies and correlates with CRS, but it has also been occasionally reported to occur independently from CRS. Early manifestations of ICANS include expressive aphasia, tremor, dysgraphia, and lethargy; these symptoms can progress to global aphasia, seizures, obtundation, stupor, and coma. ASTCT published guidelines for ICANS consensus grading based on immune effector cell-associated encephalopathy (ICE) score, depressed level of consciousness, seizure, motor findings, and cerebral edema (21). Typically, these symptoms will resolve within a week with treatment, but severe ICANS can lead to fatal intracerebral hemorrhage and malignant cerebral edema.

Scientists developed a predictive algorithm based on early fever onset and elevated IL-6 and MCP-1 serum concentrations within 36 h of CART cell infusion to identify patients at risk of developing severe ICANS. These scientists also discovered several baseline characteristics predictive of subsequent ICANS development: younger patient age, B-cell ALL, high marrow disease burden, high CART cell dose, and any pre-existing neurologic comorbidity (82). Researchers also found that elevated IL-2 and IL-5 at day 3 post-CART cell infusion were unique predictors of severe ICANS development (83). These observations may form a basis for preemptive ICANS treatment or adjustment of CART dose in high-risk patients. Most treatment centers use the anti-seizure medication levetiracetam prophylactically on the first day of infusion with CART products linked with higher ICANS incidence (84), and recently discovered predictive biomarkers have the potential to further expand prophylactic treatment protocols to reduce ICANS incidence and severity.

CART cells utilizing CD28 costimulatory domains appear to pose a greater risk for development of ICANS, although the reasons are unclear and the correlations are inconclusive (81). Efforts to develop one CD28-costimulated CART19 product were abandoned due to the ICANS-related deaths of five adult patients with ALL during the ROCKET clinical trial (85). ICANS most commonly occurs with CART19 cell therapy, and ICANS has not been observed in CART cell therapy for solid tumors to date.

### Mechanisms

Initially, there had been some concerns that CD19 expression in parts of the central nervous system contributes to the development of ICANS. ICANS can occur in patients treated with CD19-targeting bispecific antibodies as well as with CART19 cells. However, cases of ICANS have been documented with CART cell therapies targeting CD22 (86) and BCMA (87) as well as CD19, making it unlikely that ICANS occurrence is due to the nature of the target antigen and suggests an alternate mechanism. Additionally, a non-human primate study found that ICANS development was not CD19 antigen-specific, as CD20-targeted CART cells also led to ICANS in rhesus macaques (88). This study also found a marked accumulation of both CART and endogenous T cells within the cerebrospinal fluid and brain during ICANS. However, based on clinical trial data, CART cell infiltration in the central nervous system has not been found to correlate with ICANS (81, 89).

There is increasing evidence that myeloid cells have a role in the development of ICANS after CART cell administration. Based on data from mouse models, monocyte-derived IL-1 appeared to mediate ICANS as well as CRS (57), and GM-CSF-mediated stimulation of monocytes after CART cell treatment was linked to neuroinflammation in mice (62). Non-human primate models of ICANS have also shown high levels of IL-6 and GM-CSF in the cerebrospinal fluid after CART cell administration (88). In the clinic, myeloid cell-derived cytokines, most notably IL-1 and IL-6, have been shown to drive systemic inflammation which correlates with severe ICANS development (82). GM-CSF was the cytokine most significantly associated with the development of ICANS after CART cell therapy in the ZUMA-1 clinical trial (10). Analysis of the cerebrospinal fluid of patients who developed severe ICANS showed a 17-fold increase in the infiltration of CD14+ myeloid cells compared to patients with low-grade neurotoxicity (90). Additionally, patients experiencing severe ICANS often display elevated levels of the NMDA receptor agonist quinolinic acid, which is produced by stimulated macrophages. High concentrations of MCP-1, IP-10, IL-6, and IL-8 found during severe ICANS are indicative of activated macrophages and microglia (81). Collectively, these data indicate that myeloid cell hyperactivation contributes significantly to the development of ICANS.

Myeloid cell-driven inflammation may lead to endothelial activation, systemic capillary leak, and subsequent dysfunction of the blood-brain barrier frequently observed in patients with ICANS. Aberrant macrophages were found clustered around brain vasculature in a case study of fatal CART-associated ICANS (91). In another analysis, patients with severe ICANS had elevated serum concentrations of von Willebrand factor and Ang-2, which are released when endothelial cells are activated by inflammatory cytokines. Pericytes exposed to CART-generated inflammatory cytokines such as IFNγ can also produce IL-6 and VEGF, further contributing to blood-brain barrier disruption and allowing the cerebrospinal fluid to be exposed to systemic cytokines (82). It appears that endothelial activation occurs shortly after CART cell administration and precedes ICANS.

### Treatments

The standard of care for ICANS includes supportive care and the administration of corticosteroids. Paradoxically, treatment with tocilizumab may worsen ICANS symptoms. Cohort 3 of the Zuma-1 trial treated patients with tocilizumab prophylactically in combination with CART19. While this resulted in a reduction in severe CRS, there was a trend toward increased ICANS rates and severity (90). Tocilizumab is a monoclonal antibody that cannot cross the blood-brain barrier, and it blocks IL-6R in peripheral tissues that act as a systemic sink for IL-6 (20). Instead, corticosteroids are administered to treat ICANS symptoms, and shorter, rapidly tapered doses used in more recent studies have not been shown to suppress CART cell therapeutic response (10). Dexamethasone or methylprednisolone are recommended to treat moderate to severe ICANS (Grade 2+). However, there is no clinical consensus on recommendations of further treatments for patients who do not respond to high-dose steroids.

As with CRS, targeting key cytokines has shown promise in preventing or reducing ICANS after CART cell therapy. GM-CSF secretion contributes to pro-inflammatory myeloid cells, which are associated with ICANS occurrence. Not only did GM-CSF neutralization dampen CRS, but it also resulted in decreased myeloid and T-cell infiltration in the central nervous system and reduction of neuroinflammation in a patient-derived xenograft model. IL-1 secreted by activated myeloid cells has been associated with ICANS. In addition to preventing CRS, the IL-1 antagonist anakinra abolished ICANS symptoms in mice treated with CART cells (57) and is being evaluated in the clinic.

There is strong evidence of the role of endothelial activation and subsequent blood-brain barrier disruption during the development of ICANS. As such, some researchers have considered protecting endothelial cells with defibrotide, an FDA-approved drug for the treatment of hepatic veno-occlusive disease (92). While there is currently no preclinical data available on the effects of defibrotide on CART-related toxicities, a clinical trial has been initiated to investigate the prevention of ICANS with defibrotide in patients receiving axicabtagene ciloleucel.

## Additional Methods to Enhance CART Safety

Significant progress has also been made in creating safer CART cells through remote control of CART cell activation or apoptosis. Suicide genes, such as inducible caspase 9, trigger apoptotic pathways upon pharmacological activation and induce rapid and precise apoptosis in CART cells expressing the transgene (93, 94). Similarly, selection markers expressed on the CART cell surface allow CART cell elimination after administration of a neutralizing antibody, such as cetixumab for truncated EGFR-expressing CART cells (95), or rituximab for CD20-expressing CART cells (96). These methods have been explored to ablate CART cells in the event of severe toxicity, including CRS or ICANS, and clinical trials are ongoing. However, these are largely permanent, one-time approaches that will likely nullify the therapeutic effects of CART cells, although more recent studies have demonstrated dose-dependent rather than binary triggering of suicide genes (97).

It is desirable to have tunable and reversible pharmacological inhibition of CART cells in the event of severe toxicities. Researchers have shown that dasatinib, an FDA-approved tyrosine kinase inhibitor, can serve as a switch for the reversible inhibition of CART cell activity in the event of toxicities. Dasatinib temporarily suppresses T-cell activation by inhibiting T-cell receptor signaling kinases, which are also used for signal transduction in CART cells. *In vitro*, CART cells displayed a dose-dependent decrease in activation and degranulation markers, target cell killing, and cytokine secretion when dasatinib was added to cell cocultures, but regained antitumor functions within hours of dasatinib removal. Similarly, mice treated with dasatinib and CART cells displayed a reduction in CART cell function and tumor control which was restored when dasatinib administration ceased. Furthermore, these mice had significantly lower serum levels of CRS-linked inflammatory cytokines. These studies showed that dasatinib inhibition of CART cell function is dose-dependent and reversible, presenting possibilities for more precise control over CART cell activation over the course of treatment (98). Dasatinib was further tested *in vitro* and reversibly halted CART cell activity shortly after CART cell stimulation, even after sequential interactions with target cells. In a mouse model of CRS, dasatinib lowered levels of inflammatory cytokines produced by both CART cells and host myeloid cells, and mice receiving dasatinib had significantly prolonged survival (99). Additionally, dasatinib crosses the blood-brain barrier and has potential to mitigate ICANS as well as CRS. A rapid, reversible, titratable method to control CART cell activation is an appealing prospect both in the management of CART cell toxicities and in preventing CART cell exhaustion, and clinical trials are needed to further validate these findings in patients.

## Summary and Conclusion

CART cell therapy has yielded impressive outcomes in treating relapsed/refractory B-cell malignancies and has exploded with the field of immunotherapy in the last decade. However, severe and occasionally lethal toxicities, especially CRS and ICANS, remain serious issues in the clinic. As the mechanisms behind these toxicities are unraveled, improved treatment strategies can be formulated. Mounting evidence points to an important role of myeloid cells in CRS and ICANS. Many of the main inflammatory cytokines contributing to these toxicities, such as IL-1 and IL-6, appear to be primarily released by monocytes and macrophages. As such, many newer treatment strategies for CRS and ICANS are aimed at reducing myeloid cell activation. In preclinical models, GM-CSF neutralization reduced myeloid cell-derived inflammatory cytokines and ameliorated CRS and ICANS, and IL-1 inhibition reduced macrophage activity and abolished CRS and ICANS symptoms. Toxicity management strategies targeting T-cells directly have also gained momentum. JAK/STAT inhibitors decrease CRS-associated cytokines and symptoms in mice, and ITK inhibitors lengthen survival in mice and improve clinical outcomes in patients, with reduced incidence of CRS. Additionally, preventing ICANS by protecting endothelial cells with defibrotide during CART cell therapy is currently being tested in the clinic. More precise pharmacological control over CART cell activation has also gained traction in preclinical studies.

Adverse events associated with CART cell therapy, particularly CRS and ICANS, are complex and have overlapping features. Further distillation of the mechanisms behind these toxicities and identification of predictive biomarkers have provided more insight and inspiration for novel management and prevention methods. Ideally, these strategies will become preventative rather than reactive, leading to simplified clinical management, widespread implementation of CART cell therapy outside of specialized treatment centers, reduction of medical and financial burden, and improved patient outcomes.

## Author Contributions

ES wrote the initial manuscript. ES and SK edited and approved the final manuscript. Both authors contributed to the article and approved the submitted version.

## Conflict of Interest

SK is an inventor on patents in the field of CAR immunotherapy that are licensed to Novartis (through an agreement between Mayo Clinic, University of Pennsylvania, and Novartis), and patents in the field of CAR immunotherapy that are licensed to Humanigen and Mettaforge (through Mayo Clinic). SK receives research funding from Kite, Gilead, Juno, Celgene, Novartis, Humanigen, MorphoSys, Tolero, Sunesis, and Lentigen. The remaining author declares that the research was conducted in the absence of any commercial or financial relationships that could be construed as a potential conflict of interest.
